# ReCell Combined With NB‐UVB for the Treatment of Extensive Stable Vitiligo: A Randomized Controlled Trial

**DOI:** 10.1111/jocd.70032

**Published:** 2025-02-12

**Authors:** Xueya Tong, Zhiming Yuan, Yu Lei

**Affiliations:** ^1^ Chengdu Borun Vitiligo Hospital Dermatology Chengdu China; ^2^ School of Pharmacy China Medical University Shenyang China

**Keywords:** phototherapy, surgery, vitiligo

To the Editor,

Vitiligo is a chronic autoimmune disease characterized by skin depigmentation, affecting 0.5%–2% of the global population [1]. Patients often receive internal medications combined with local or systemic interventions, such as phototherapy, to regulate systemic immunity, improve the microenvironment of white spots, and inhibit lesion expansion [2]. Surgery is commonly employed for treating stable vitiligo [3]. Surgical procedures, such as skin grafts, microdrill grafts, and negative‐pressure blister suction epidermal grafts, are typically restricted to smaller lesions and may cause cobblestone‐like hyperpigmentation at the donor site [4]. ReCell is an epidermal cell transplantation technique used to treat stable, large‐area vitiligo. It facilitates rapid regeneration and reepithelialization of the epidermis with an area expansion ratio of 20:1–80:1, depending on the treatment site [5]. Changes in the internal environment of skin lesions are critical to the effectiveness of surgical treatment and the subsequent repigmentation process. Narrow‐band ultraviolet B (NB‐UVB), a first‐line phototherapy for vitiligo, supports surgical interventions by stimulating melanocyte proliferation, suppressing T lymphocytes, and inhibiting cytokines to create a favorable environment for transplanted melanocytes [6]. This study assessed the clinical efficacy of combining ReCell and NB‐UVB in vitiligo patients.

The Ethics Committee of Chengdu Borun Vitiligo Hospital approved this study (2024BR‐087‐01) (Appendix S1). Written informed consent was obtained from all patients. Inclusion criteria included: ① meeting the diagnostic criteria for stable vitiligo as defined in the Vitiligo Diagnosis and Treatment Consensus (2021 edition) [3], ② no history of local or systemic glucocorticoid use within the past 6 months, and ③ a surgical treatment area exceeding 200 cm^2^ in a single session. Criteria for stable vitiligo included: ① a vitiligo disease activity (VIDA) score of 0; ② clinical features such as porcelain‐white spots with clear edges or residual pigmentation; ③ absence of isomorphic reactions for ≥ 1 year; and ④ under a Wood lamp, the affected skin area appeared smaller than the visually observed area. A total of 46 patients (21 males, 25 females; mean age: 23 years; age range: 15–48 years) were enrolled in the study. Patients were randomly assigned into two groups, with 23 patients in each. Both groups received ReCell treatment. The treatment group additionally underwent NB‐UVB (Waldmann Company, Germany) irradiation starting 2 weeks postsurgery, at a frequency of twice per week. The initial dose was 300–400 mJ/cm^2^, with a 20% increment per treatment, for a total of 30 treatments [7].

Patients were followed up at 3 and 6 months postoperatively. Pigmentation was evaluated using photographs taken under a Wood's lamp, where fluorescent areas indicated vitiligo or hypopigmented skin, and nonfluorescent areas represented normal or hyperpigmented skin. A single surgeon calculated the fluorescent area three times for each follow‐up photograph using ImageJ (National Institutes of Health) to determine an average value. Repigmentation rate = (repigmented area at each follow‐up visit)/(baseline vitiligo area). Repigmentation was graded as excellent (> 75%), good (50%–75%), moderate (25%–50%), or poor (≤ 25%) based on repigmentation rates. Effective rate [8] = [(number of excellent repigmentation cases + number of good repigmentation cases)/total cases] × 100%. Potential adverse reactions, including scarring, infection, and recurrence, were documented to evaluate treatment safety. Recurrence was defined as the reappearance of vitiligo symptoms in previously stable or healed areas. Group data were analyzed using the chi‐squared test, with statistical significance set at *p* < 0.05.

At the 3‐month follow‐up, the treatment group demonstrated a higher effective rate (78.26%) compared to the control group (52.17%); however, this difference was not statistically significant (*p* > 0.05). At the 6‐month follow‐up, the effective rate of the treatment group (82.61%) was significantly higher than that of the control group (52.17%) (*χ* ^2^ = 4.847, *p* = 0.02769) (Figure 1, Table 1). Table 2 presents the 6‐month follow‐up comparison of lesion efficacy across different body parts between the two groups, showing no significant difference in repigmentation rate (*p* > 0.05). In the control group, postoperative adverse reactions included itching in four patients. In the treatment group, six patients experienced itching, and two developed blisters following phototherapy. Investigation revealed that itching in both groups was mild, and the blisters in the treatment group were small and localized to the lesion sites.

**FIGURE 1:**
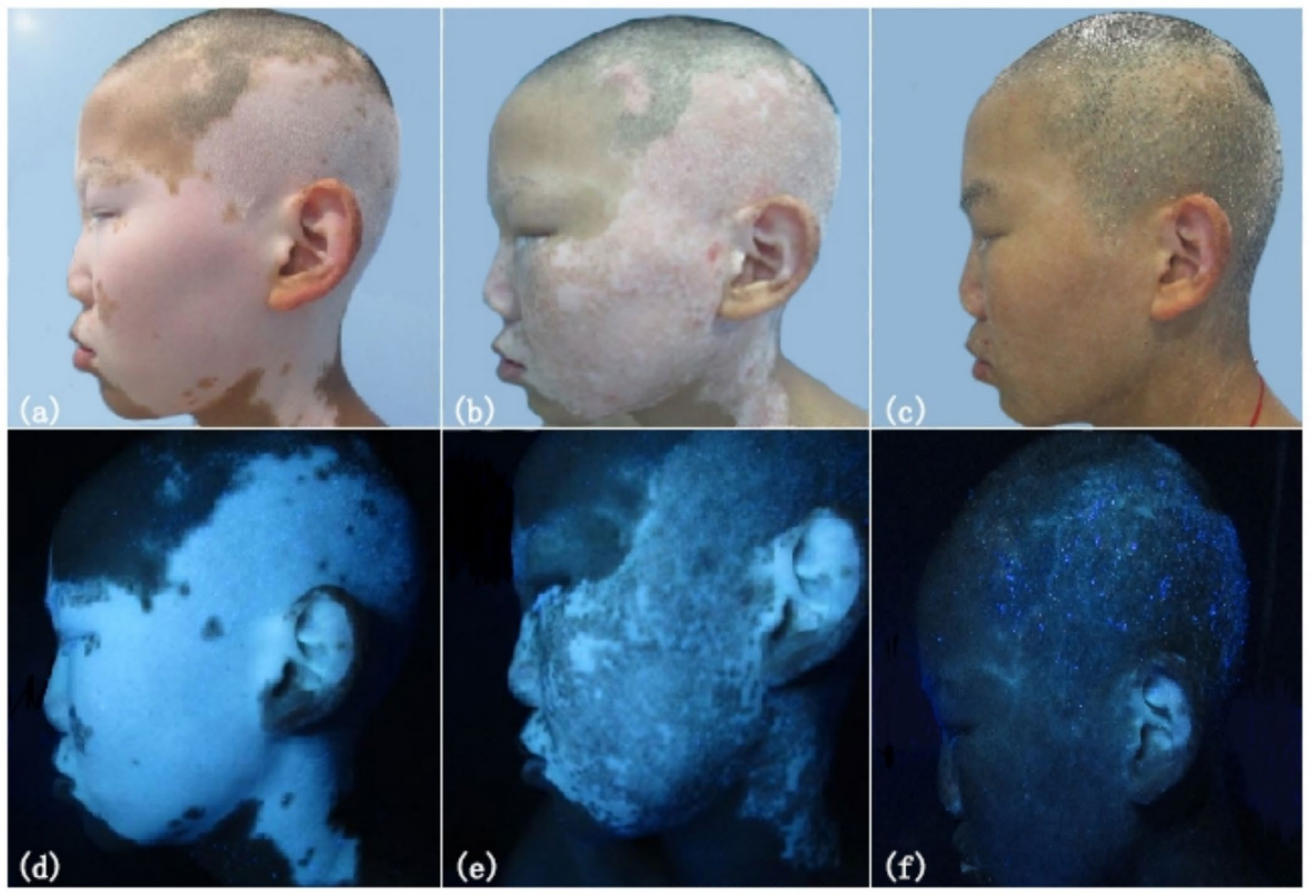
ReCell combined with NB‐UVB treatment group: (a) baseline (normal light), (b) 3 months (normal light), (c) 6 months (normal light), (d) baseline (Wood lamp), (e) 3 months (Wood lamp), (f) 6 months (Wood lamp).

**TABLE 1:** Comparison of the efficacy of the two groups of patients at the third and sixth months of follow‐up.

Follow‐up time	Group	Repigmentation grade				Efficacy rate (%)
		Exellent	Good	Medium	Bad	
Third mouth	NB‐UVB	6	6	6	5	52.17
	Recell + NB‐UVB	10	8	3	2	78.26
Sixth mouth	NB‐UVB	7	5	7	4	52.17
	Recell + NB‐UVB	14	5	3	1	82.61

**TABLE 2:** Comparison of the efficacy of various parts of the body in the two groups at the sixth month of follow‐up.

Repigmentation grade	Neck		Trunk		Extremity	
	NB‐UVB	Recell + NB‐UVB	NB‐UVB	Recell + NB‐UVB	NB‐UVB	Recell + NB‐UVB
Exellent	4	7	2	4	1	3
Good	3	1	1	1	1	2
Medium	1	1	4	1	2	2
Bad	1	0	1	1	2	0
Efficacy rate (%)	77.8	88.9	37.5	71.4	33.3	71.4

The overall efficacy of ReCell treatment reported in previous studies was lower than that observed with the combination of ReCell and NB‐UVB in this study. Mulekar et al. reported that at the 4‐month follow‐up, ReCell treatment achieved a 60% efficacy rate across five lesions (two lesions showed 100% repigmentation, one 65%, one 40%, and one did not repigment) [9]. Luo et al. [10] treated 31 patients with ReCell, achieving an efficacy rate of 80.64% at the 6‐month follow‐up (25 patients demonstrated a repigmentation rate of ≥ 50%). In this study, the superior efficacy observed in the treatment group compared to the control group may be attributed to the postoperative NB‐UVB therapy. The repigmentation mechanism of NB‐UVB involves several processes: it promotes melanin synthesis by stimulating the proliferation, differentiation, and migration of pigment‐free melanocytes in the outer follicular root sheath, melanogenic stem cells, and residual melanocytes in leukoplakia, while also enhancing intracellular tyrosinase activity [11]. Second, NB‐UVB induces apoptosis in pathogenic T‐lymphocytes and keratinocytes, thereby mitigating epidermal hyperproliferation and exerting local and systemic immunosuppressive effects [12]. Additionally, NB‐UVB restores the Th17/Tregs balance and enhances the microenvironment of the depigmented areas [13]. Overall, the application of NB‐UVB following ReCell leads to enhanced repigmentation results; however, potential adverse effects of postoperative NB‐UVB therapy should be carefully monitored.

## Conflicts of Interest

The authors declare no conflicts of interest.

## Supporting information


Appendix S1.

## Data Availability

The authors have nothing to report.
